# Visual field prediction using a deep bidirectional gated recurrent unit network model

**DOI:** 10.1038/s41598-023-37360-1

**Published:** 2023-07-10

**Authors:** Hwayeong Kim, Jiwoong Lee, Sangwoo Moon, Sangil Kim, Taehyeong Kim, Sang Wook Jin, Jung Lim Kim, Jonghoon Shin, Seung Uk Lee, Geunsoo Jang, Yuanmeng Hu, Jeong Rye Park

**Affiliations:** 1 Department of Ophthalmology, Pusan National University College of Medicine, Busan, Korea; 2 Biomedical Research Institute, Pusan National University Hospital, Busan, Korea; 3 Department of Mathematics, Pusan National University, Busan, Republic of Korea; 4 Department of Ophthalmology, Dong-A University College of Medicine, Busan, Korea; 5 Department of Ophthalmology, Busan Paik Hospital, Inje University College of Medicine, Busan, Korea; 6 Department of Ophthalmology, Pusan National University Yangsan Hospital, Pusan National University School of Medicine, Yangsan, Korea; 7 Department of Ophthalmology, Kosin University College of Medicine, Busan, Korea; 8 Nonlinear Dynamics and Mathematical Application Center, Kyungpook National University, Daegu, Korea; 9 Department of Mathematics, Kyungpook National University, 80, Daehak-ro, Buk-gu, Daegu, 41566 Republic of Korea

**Keywords:** Medical research, Mathematics and computing

## Abstract

Although deep learning architecture has been used to process sequential data, only a few studies have explored the usefulness of deep learning algorithms to detect glaucoma progression. Here, we proposed a bidirectional gated recurrent unit (Bi-GRU) algorithm to predict visual field loss. In total, 5413 eyes from 3321 patients were included in the training set, whereas 1272 eyes from 1272 patients were included in the test set. Data from five consecutive visual field examinations were used as input; the sixth visual field examinations were compared with predictions by the Bi-GRU. The performance of Bi-GRU was compared with the performances of conventional linear regression (LR) and long short-term memory (LSTM) algorithms. Overall prediction error was significantly lower for Bi-GRU than for LR and LSTM algorithms. In pointwise prediction, Bi-GRU showed the lowest prediction error among the three models in most test locations. Furthermore, Bi-GRU was the least affected model in terms of worsening reliability indices and glaucoma severity. Accurate prediction of visual field loss using the Bi-GRU algorithm may facilitate decision-making regarding the treatment of patients with glaucoma.

## Introduction

Glaucoma, a leading cause of blindness worldwide, is characterized by irreversible loss of retinal ganglion cells^1,2^. Structural changes in retinal ganglion cells and the optic nerve head cause progressive deterioration of the visual field^2^. The prediction of future visual field is essential to preserve visual function. However, visual field test results are susceptible to random errors and fluctuations, particularly in patients with glaucoma, which hinders accurate prediction of visual field changes^3^.

Over the past several years, machine learning algorithms have demonstrated good performance in the prediction of glaucoma progression. Wang et al.^4^ classified and determined the progression of 16 archetypes of visual field defects. Murata et al.^5^ found superior prediction ability of variational Bayes linear regression, a type of machine learning algorithm, compared with pointwise linear regression (LR). Because of the recent development of artificial intelligence, deep learning algorithms have been used for various tasks with excellent performance. However, only a few studies have predicted the progression of visual field defects using deep learning algorithms. Wen et al.^6^ used a convolutional neural network to predict future visual fields, using a single visual field examination as input. Berchuck et al.^7^ used a variational autoencoder model to estimate the rate of visual field progression.

Recurrent neural network (RNN), an artificial network with recurrent connections, has been used for sequential time series with temporal dependence and for sequence modeling^8^. It can process current data, using previous data to make predictions, based on dependencies between sequential elements^9,10^. The two main variants of RNN, long short-term memory (LSTM)^11^ and gated recurrent unit (GRU)^12^, model long-term dependency into long sequences. In a previous study, we found that LSTM had superior abilities to predict future visual fields, compared with ordinary least-squares LR^13^. Dixit et al.^14^ found that LSTM networks can predict the longitudinal local and global trends in visual fields.

GRU uses gating units more efficiently and at a similar rate, compared with typical LSTMs^15–17^. Several studies have revealed that GRU has excellent performance for sequential data analysis, compared with other RNN types^12,15,18,19^. Recently, a bidirectional RNN method has been developed via simultaneous training with positive and negative time directions, which provides a better understanding of context^20^. Lynn et al.^15^ compared several RNN-based models for human identification using electrocardiogram-based biometrics from sequential time-series data. The bidirectional network with LSTM and GRU models was more effective than conventional RNN models, and the bidirectional-gated recurrent unit (Bi-GRU) model exhibited performance superior to the bidirectional LSTM model. Because visual field examinations provide sequential data with extensive interconnections, Bi-GRU may achieve better prediction of visual field progression, compared with the previous LSTM-based RNN model.

To our knowledge, this is the first study to use Bi-GRU to predict visual field damage. In a previous study, we evaluated the performance of LSTM in predicting visual field defects. Because the present study used a larger dataset than our previous work, we developed a computationally efficient RNN-based Bi-GRU model. We compared the performance of the Bi-GRU model with the performances of conventional LR and LSTM models.

## Materials and methods

This retrospective study was conducted in accordance with the tenets of the Declaration of Helsinki. Visual field data were collected from glaucoma clinics at Pusan National University Hospital, Kosin University Gospel Hospital, Dong-A University Hospital, Busan Paik Hospital, and Pusan National University Yangsan Hospital between June 2004 and January 2021. The study protocol was approved by the institutional review boards of Pusan National University Hospital (Approval No.: 2203-018-113), Kosin University Gospel Hospital (Approval No.: 2018-12-028), Dong-A University Hospital (Approval No.: 22-074), Busan Paik Hospital (Approval No.: 2021-03-014-002), and Pusan National University Yangsan Hospital (Approval No.: 05-2018-172). The requirement for patient consent was waived by the institutional review boards because of the retrospective study design. Sex and diagnostic data were retrospectively collected from medical records.

**Figure 1:**
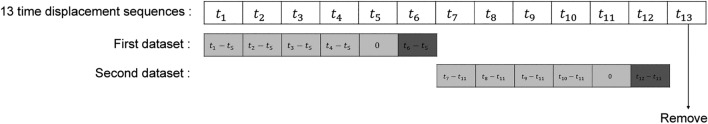
Representative time displacement sequence of a patient who completed 13 visual field tests. Visual field test dates indicated in gray boxes were used for training, and dates in black boxes were used for prediction.

Participants who completed a minimum of six consecutive visual field examinations were included in the training and test datasets. There was no patient overlap between the two datasets. Eyes with an interval of ≥ 3 years between the first and sixth visual field examinations were included. For example, in an eye with 13 consecutive visual field examinations, the first through sixth examinations were considered the first dataset, the seventh through twelfth examinations were considered the second dataset, and the thirteenth examination was excluded from the dataset. The first five examinations were used as input data to predict the sixth examination, and the seventh through eleventh examinations were used as input data to predict the twelfth examination (Fig. 1).

We obtained 6-cell data from 8323 visual fields of 6685 eyes and 4593 participants. Datasets from 7051 (85%) and 1,272 (15%) individuals were included in the training and test datasets, respectively. In total, 7051 records from the training dataset were randomly split into training and validation datasets at a ratio of 9:1. The validation dataset was used to determine the fitness of the neural network during training to prevent overfitting. All 8323 datasets included six visual field examinations, and the mean follow-up duration for the six examinations was 4.39 $\pm$ 1.69 years. Table 1 presents the characteristics of each dataset.

**Table 1:** Demographic characteristics.

Demographic characteristics	All data	Training data	Test data
Total number of eyes (patients)	6685 (4593)	5413 (3321)	1272 (1272)
Age (years), mean $\pm$ SD	53.96 $\pm$ 16.04	54.11 $\pm$ 15.88	53.13 $\pm$ 16.85
Initial visual field MD (dB), mean $\pm$ SD	− 5.83 $\pm$ 6.21	− 5.77 $\pm$ 6.16	− 6.19 $\pm$ 6.44
Follow-up duration (years), mean $\pm$ SD	5.63 $\pm$ 2.75	5.87 $\pm$ 2.87	4.61 $\pm$ 1.84
Mean number of visual field tests	7.47 $\pm$ 3.08	7.82 $\pm$ 3.33	6.00 $\pm$ 0.00
MD $\ge -$ 6 dB	4415	3587	828
$-$ 6 dB $>$ MD $\ge$ $-$ 12 dB	1217	981	236
$-$ 12 dB $>$ MD	1053	845	208
Data augmentation			
Total number of datasets with 6 in a pair	8323	7051	1272
Follow-up duration (years), mean $\pm$ SD	4.39 $\pm$ 1.69	4.35 $\pm$ 1.66	4.61 $\pm$ 1.84
Prediction time interval (years), mean $\pm$ SD	0.94 $\pm$ 0.73	0.92 $\pm$ 0.71	1.00 $\pm$ 0.84
MD $\ge -$ 6 dB	5579	4751	828
$-$ 6 dB $>$ MD $\ge$ $-$ 12 dB	1477	1241	236
$-$ 12 dB $<$ MD	1267	1059	208

*MD* mean deviation, *SD* standard deviation.

### Visual field examination

Automated perimetry was conducted using a Humphrey Visual Field Analyzer 750i (Carl Zeiss Meditec, Inc., Dublin, CA, USA) and the 24-2 or 30-2 Swedish interactive threshold algorithm. Among the 54 test points of the 24-2 test pattern, the two points of physiological scotoma were excluded; the remaining 52 test points were used. The 30-2 test pattern was converted to the 24-2 test pattern using the overlapped test points. Reliable visual field tests were defined as a false positive rate < 33%, false negative rate < 33%, and fixation loss < 33%.

### Artificial neural network

We used the LSTM and Bi-GRU neural network models. Python software (version 3.8) with TensorFlow 2.3 (Google, Mountain View, CA, USA) was used to predict visual field loss. Supplementary Fig. S1 illustrates the two model structures.

#### LSTM and Bi-GRU

We built one-layer neural networks to learn the structural information of a specific dataset using preprocessed input. The LSTM cell-based neural networks were defined as follows:

1 $$F_{orgetgate} = sigmoid\left( {W_{f} X_{t} + W_{hf} h_{t - 1} + b_{f} } \right)$$

2 $$I_{nputgate} = sigmoid\left( {W_{i} X_{t} + W_{hi} h_{t - 1} + b_{i} } \right)$$

3 $$O_{utputgate} = sigmoid\left( {W_{o} X_{t} + W_{ho} h_{t - 1} + b_{o} } \right)$$

4 $$\left( C \right)_{t} = \left( C \right)_{t - 1} \otimes \left( {F_{orgetgate} } \right)_{t} + \left( {I_{nputgate} } \right)_{t} \otimes \left( {\tanh \left( {W_{C} X_{t} + W_{hC} h_{t - 1} } \right) + b_{C} } \right)$$

5 $$h_{t} = O_{utputgate} \otimes \tanh \left( {\left( C \right)_{t - 1} } \right)$$

where ${W}_{f},{W}_{i}, {W}_{o}, and {W}_{C}$ represent the weights and ${b}_{f}, {b}_{i}, {b}_{o}, { and b}_{C}$ represent the bias in the network, respectively, of the three gates and a memory cell. ⨂ is the elementwise product between two vectors. The sigmoid is the activation function used in the network, written as follows:

$$sigmoid\left( x \right) = \frac{1}{{1 + e^{ - x} }}$$

The input and output gates regulate the flow of memory cell inputs and outputs throughout the network, while the forget gate is incorporated into the memory cell to transmit output information with high weights from the previous neuron to the next one. The information residing in the memory depends on the high activation results. If the input unit has high activation, information is stored in the memory cell. On the other hand, if the output unit has high activation, it passes the information to the next neuron. Input information with a high weight resides in the memory cell. Sigmoid and tanh are employed as the active functions for the gates. Here, h(t-1) represents the prior hidden layer units that add the weights of the three gates in an elementwise manner. After processing Eq. (4), (C)_t_ indicates the current memory cell unit. Equation (5) shows the elementwise multiplication of the prior hidden unit outputs and previous memory cell unit. Nonlinearity is introduced through the tanh and sigmoid activation functions as shown in Eqs. (1–5). Here, *t* − 1 and t are the previous and current time steps.

GRU is a simplified variant of LSTM that only has two gates: the update gate, which comprises the input and forget gates, and the reset gate. It has no additional memory cell to retain information and can only control information inside the unit.

6 $$U_{pdategate} = sigmoid\left( {W_{u} X_{t} + W_{hu} h_{t - 1} + b_{u} } \right)$$

7 $$R_{estgate} = sigmoid\left( {W_{r} X_{t} + W_{hr} h_{t - 1} + b_{r} } \right)$$

8 $$\widetilde{{h_{t} }} = tanh\left( {WX_{t} + W\left( {R_{estgate} \otimes h_{t - 1} } \right)} \right)$$

9 $$h_{t} = \left( {1 - \left( {U_{pdategate} } \right)_{t} } \right) \otimes h_{t - 1} + \left( {U_{pdategate} } \right)_{t} \otimes h_{t}$$

The update gate in Eq. (6) determines the extent of information updating. In Eq. (7), the rest gate is similar to the update gate; if the gate is set to zero, GRU reads the input sequences and forgets the previously calculated state. Furthermore, $\widetilde{{h}_{t}}$ exhibits functionality identical to the recurrent unit, and ℎ_*t*_ of the GRU at time t represents linear interpolation among the current $\widetilde{{h}_{t}}$ and previous ${h}_{t-1}$ activation states in Eqs. (8) and (9).

A Bi-GRU layer was formed by combining a forward GRU with a reverse-direction GRU. Both GRUs receive the same input but train in opposite directions, and their results are concatenated to produce the output. Deep hierarchical neural networks effectively capture specific functions and model dependencies of varying lengths^21^. Our experiments revealed that Bi-GRU outperformed other models on our datasets.

#### Proposed method and evaluation

**Figure 2:**
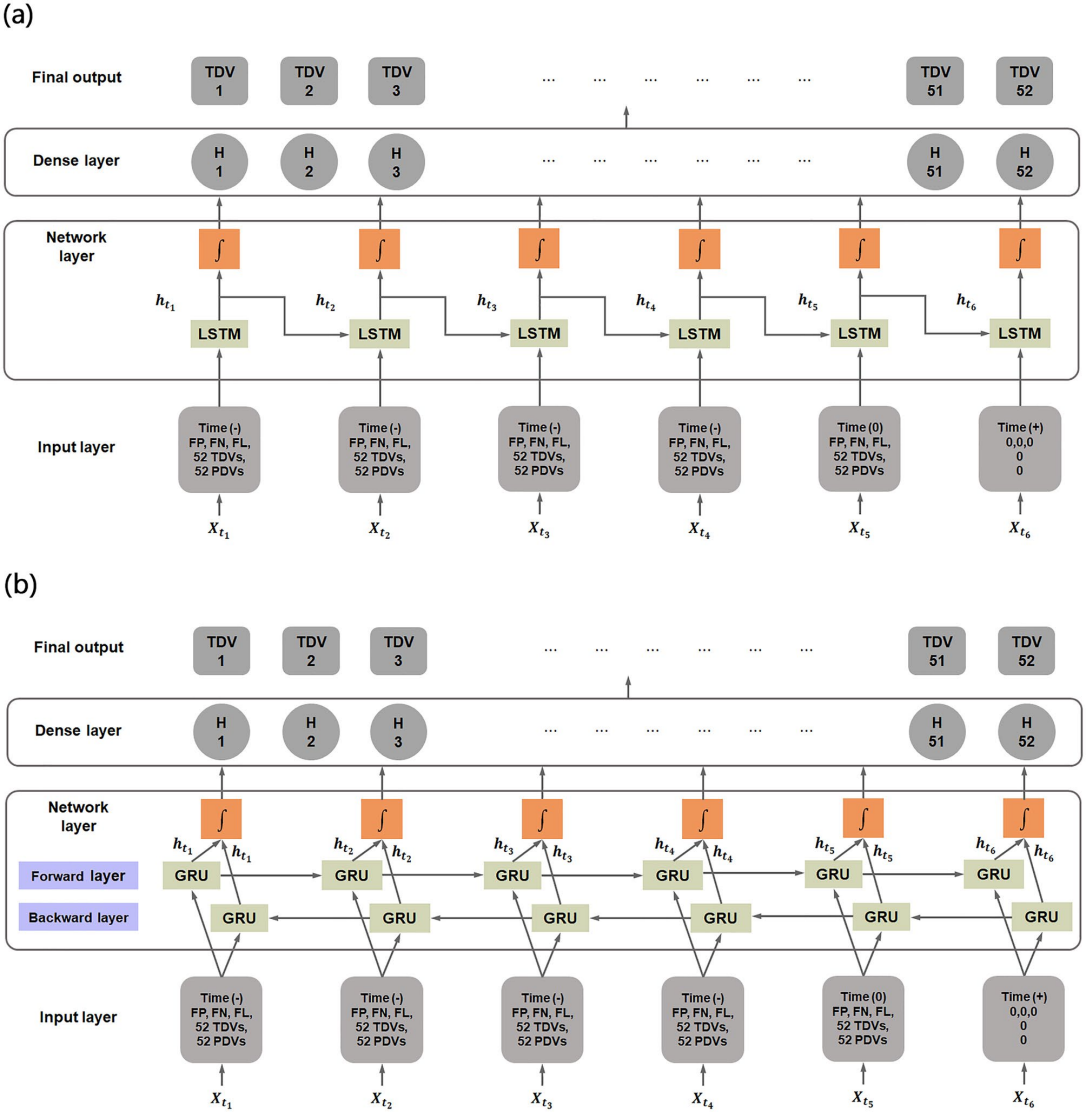
Architectures of the (**a**) long short-term memory (LSTM) method and (**b**) bidirectional gated recurrent unit (Bi-GRU) method. The input layers of both models consisted of time displacement values in days, reliability data, and visual field data. Reliability data consisted of false-positive (FP) rate, false-negative (FN) rate, and fixation loss (FL) percentage. Visual field data consisted of 52 pattern deviation values (PDVs) and 52 total deviation values (TDVs) on the 24-2 visual field test (two points of physiological scotoma were excluded). The last cell contained a positive time displacement value and 107 zeros as input because all other values were set to zero. These unique inputs can specify the exact date the user wants to predict. LSTM = long short-term memory; Bi-GRU = bidirectional gated recurrent unit; TDV = total deviation value.

In our proposed method, the deep learning model comprises input data, a one-time series neural network layer used for sequential predictions, and a dense layer. The neural network structures for LSTM and Bi-GRU are shown in Fig. 2.

The single-layer time-series neural network consists of six parallel and connected LSTM or Bi-GRU cells. The detailed structures of the LSTM and GRU cells are presented in Supplementary Fig. S1a, b, respectively.

Each of the first five cells uses 108 features as input, including 52 total deviation values (TDVs), 52 pattern deviation values (PDVs), reliability data (such as false-negative and false-positive rates, fixation loss percentage), and time displacement value. To improve the performance of the deep learning model, the input data were normalized to a reasonable range. The TDV, PDV, and time displacement values were divided into sets of 50, 50, and 1000, respectively. Time displacement indicated the number of days from the most recent visual field examination. For example, if the most recent visual field examination has a time displacement of “0,” the visual field examination performed 1 month (− 31 days) prior to “0” has a time displacement of “ − 31.” A negative sign in the time displacement value indicates that the examination was performed in the past. With respect to the 6 consecutive visual field input data elements, the last input data element used a unique format with positive time displacement (i.e., the point in the future that the user wishes to predict) and 107 zeros. Since the other data were set to 0, these unique inputs can specify the exact date which the user wishes to predict. A series of input data was arranged by reducing the time displacement value (i.e., from future to past) and then supplying this information to the neural network. Subsequently, the neural network layer was connected to the next single fully connected layer (dense layer) with 52 neurons. These neurons generated a final output of 52 TDVs, such that one neuron generated a single visual field test point.

### Statistical analyses

The root mean square error (RMSE) and mean absolute error (MAE) of the TDV were used as accuracy metrics. The RMSE was calculated for each eye using the following equation:

$$\begin{aligned} RMSE & = \sqrt {\mathop \sum \limits_{n = 1}^{52} \frac{{\left( {true\; TDV_{n} - predicted \;TDV_{n} } \right)^{2} }}{52}} \\ n & = nth\; test\;point\;of\;visual\; field\; exam \\ \end{aligned}$$

The MAE was calculated for each test point in the visual field of all eyes using the following equation:

$$\begin{aligned} MAE_{n} & = \mathop \sum \limits_{i = 1}^{number\; of\; eyes} \frac{{\left| {true \;TDV_{i,n} - predicted\; TDV_{i,n} } \right|}}{number\; of\; eyes} \\ n & = nth\; test\;point\; of\; visual\; field\; exam \\ i & = ith\; eye \\ TDV_{i,n} & = total \;deviation\; value \;of \;ith\; eye,\; nth\; test\;point \\ \end{aligned}$$

The RMSE and MAE of the LR, LSTM, and Bi-GRU models were calculated using the above formulas. Repeated measures one-way analysis of variance was performed to compare accuracy metrics among LR, LSTM, and Bi-GRU models. *P* < 0.05 (single comparison) and *p* < 0.017 (multiple comparisons) were considered indicative of statistical significance. Parametric and nonparametric tests (Spearman’s correlation and simple LR analyses) were performed to compare variables. These tests were used to investigate prediction error trends according to various factors, including false positive rate, false negative rate, fixation loss percentage, and visual field mean deviation (MD).

## Results

**Figure 3:**
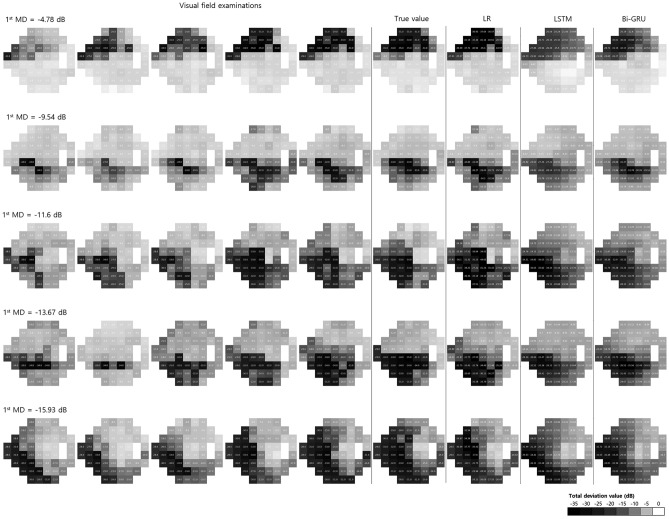
Representative examples of visual field prediction according to mean deviation (MD) of the first visual field examination. Five consecutive input visual field examinations are shown in chronological order from left to right, followed by the sixth examination (regarded as the true value). Columns 7–9 indicate the prediction results of LR, LSTM, and Bi-GRU models, respectively. LR = linear regression; LSTM = long short-term memory; Bi-GRU = bidirectional gated recurrent unit.

Table 2 shows the demographic characteristics of the test dataset. The most common diagnosis was primary open-angle glaucoma (47.68%). The mean prediction time (time interval between prediction and final visual field examination) was 1.00 ± 0.84 years (Table 1). The mean RMSE and pointwise mean absolute error (PMAE) are shown in Table 3. Figure 3 presents representative examples of the PMAE in the visual field test.

**Table 2:** Demographic characteristics of the test dataset.

	Number of eyes
Total	1271
Sex, male (%)	599 (47.13)
Diagnosis	
Glaucoma suspect	360
Primary open-angle glaucoma	606
Pseudoexfoliative glaucoma	23
Primary angle-closure glaucoma	76
Secondary glaucoma	90
Others	111

**Table 3:** Comparison of mean prediction error (RMSE and PMAE) among LR, LSTM, and Bi-GRU models.

	LR	LSTM	Bi-GRU	*p* value*	*p* value^†^		
					LR vs. Bi-GRU	LSTM vs. Bi-GRU	LR vs. LSTM
*Prediction error, mean *$\pm$* SD*							
RMSE (dB)	4.81 $\pm$ 3.89	4.06 $\pm$ 2.61	3.71 $\pm$ 2.42	< 0.001	< 0.001	< 0.001	< 0.001
PMAE (dB)	3.52 $\pm$ 0.56	3.10 $\pm$ 0.39	2.80 $\pm$ 0.36	< 0.001	< 0.001	< 0.001	< 0.001

*RMSE* root mean square error, *SD* standard deviation, *PMAE* pointwise mean absolute error, *LR* linear regression, *LSTM* long short-term memory, *Bi-GRU* bidirectional gated recurrent unit.

*Significance level ɑ = 0.05.

^†^Significance level ɑ = 0.017.

Bi-GRU exhibited better prediction performance, compared with LR and LSTM. The RMSEs of Bi-GRU, LR, and LSTM were 3.71 ± 2.42, 4.81 ± 3.89, and 4.06 ± 2.61 dB, respectively. There were statistically significant differences in prediction errors among the three models (F = 42.94, *p* < 0.001). The RMSE was significantly lower for Bi-GRU than for the other two models (both *p* < 0.001).

**Figure 4:**
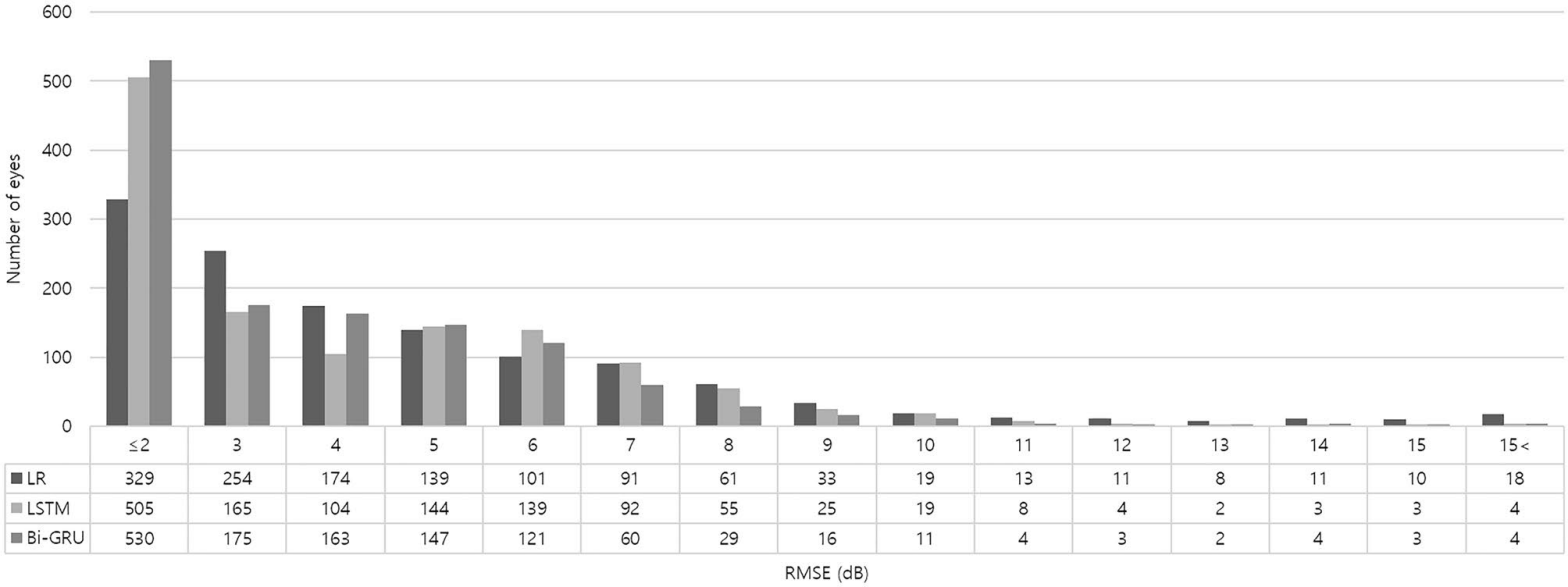
Number of eyes binned according to prediction error (RMSE, root mean squared error).

The number of eyes binned according to RMSE prediction error is shown in Fig. 4. More than 50% of eyes had Bi-GRU prediction errors of ≤ 2 dB (530 eyes, 41.67%) and 2–3 dB (175 eyes, 13.76%). The corresponding LR prediction errors were ≤ 2 dB (329 eyes, 25.86%) and 2–3 dB (254 eyes, 19.97%), and the corresponding LSTM prediction errors were ≤ 2 dB (505 eyes, 39.70%) and 2–3 dB (165 eyes, 12.97%).

**Figure 5:**
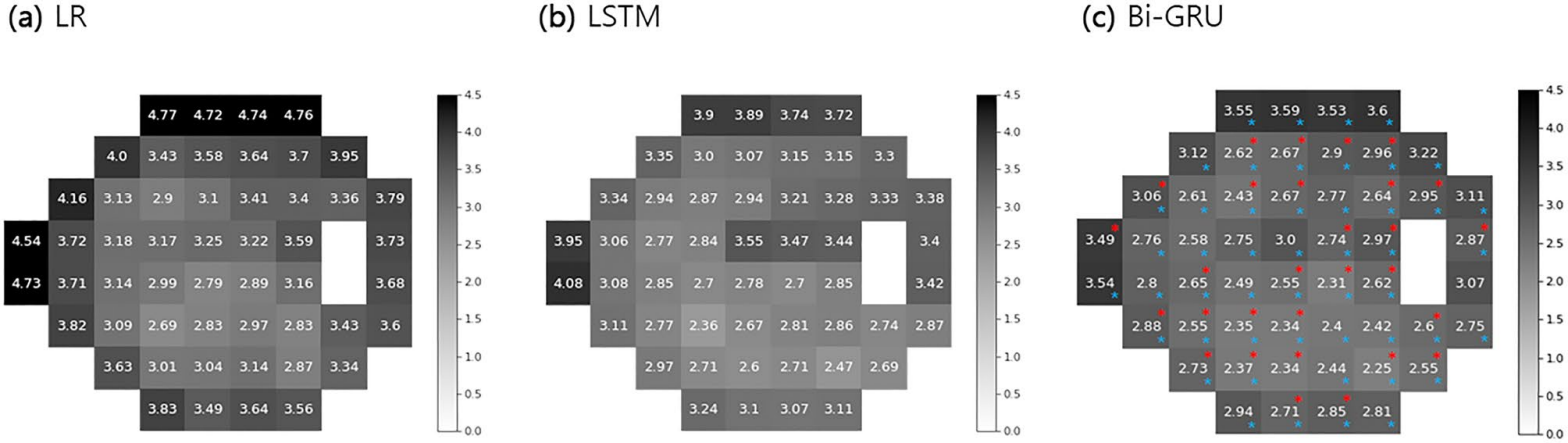
Pointwise mean absolute error (PMAE) of predicted total deviation value (TDV). Bi-GRU had the lowest prediction error (PMAE) for all 52 points. Darker colors indicate higher error. Red dots indicate significant differences between LR and Bi-GRU; blue dots indicate significant differences between LSTM and Bi-GRU (paired t-test). LR = linear regression; LSTM = long short-term memory; Bi-GRU = bidirectional gated recurrent unit.

Figure 5 shows the PMAE in the visual field. With respect to the 52 TDV points, Bi-GRU exhibited the lowest prediction error among the three models. Bi-GRU showed significantly better performance at 29 (red dots) and 49 (blue dots) points compared with LR and LSTM, respectively.

**Figure 6:**
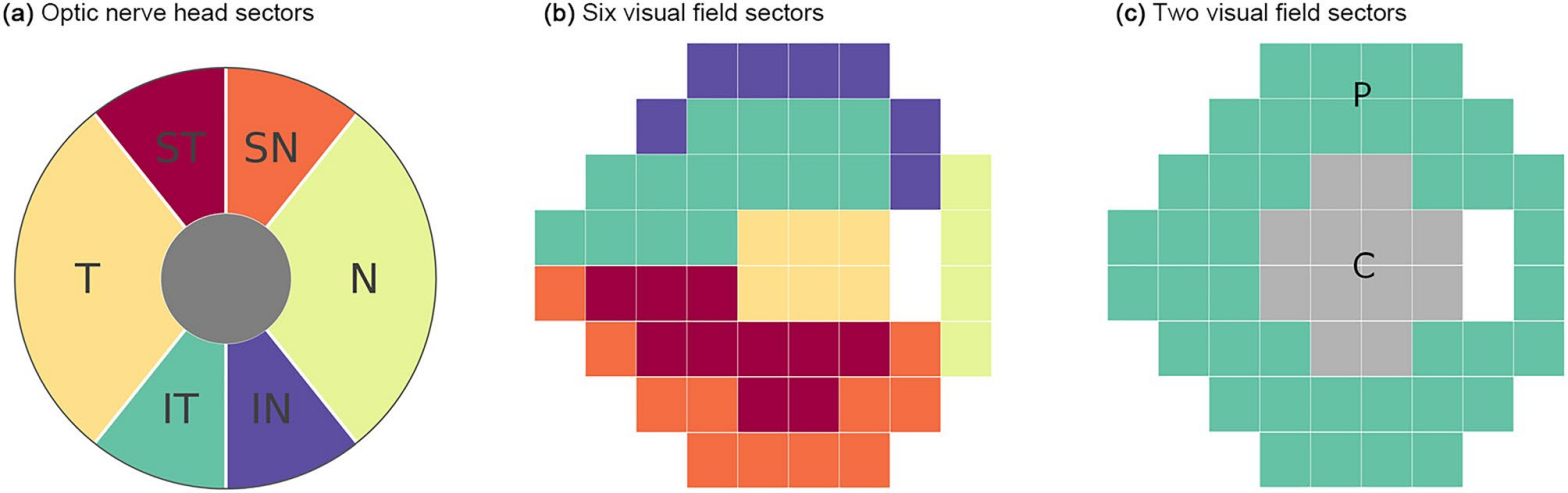
Division of the optic nerve head (**a**) and visual field (**b, c**). (**b**) The visual field was divided into six sectors proposed by Garway-Heath et al.^22^ (**c**) The visual field was divided into the central and peripheral zones. ST = superotemporal; SN = superonasal; T = temporal; N = nasal; IT = inferotemporal; IN = inferonasal; P = peripheral; C = central.

Table 4 shows the mean prediction error (RMSE) according to sectors of the visual field examination (Fig. 6). The 24-2 visual field was divided into the six sectors proposed by Garway-Heath et al.,^22^ based on optic nerve head anatomy (superotemporal, superonasal, temporal, nasal, inferotemporal, and inferonasal) [Fig. 6b] and two sectors (central and peripheral) [Fig. 6c]. The prediction errors of Bi-GRU were significantly lower than the errors of LR and LSTM for all sectors (*p* ≤ 0.001).

**Table 4:** Comparisons of mean prediction error (RMSE) among LR, LSTM, and Bi-GRU models in six Garway-Heath sectors and peripheral and central zones on the 24–2 visual field test.

	Prediction error (RMSE, dB), mean $\pm$ SD			*p* value*	*p* value^†^		
	LR	LSTM	Bi-GRU		Bi-GRU vs. LSTM	Bi-GRU vs. LR	LR vs. LSTM
Superotemporal	3.84 $\pm$ 4.08	3.29 $\pm$ 2.86	3.02 $\pm$ 2.55	< 0.001	< 0.001	< 0.001	< 0.001
Superonasal	4.55 $\pm$ 4.18	3.71 $\pm$ 2.76	3.41 $\pm$ 2.56	< 0.001	< 0.001	< 0.001	< 0.001
Temporal	3.93 $\pm$ 4.52	3.79 $\pm$ 3.52	3.28 $\pm$ 2.91	< 0.001	< 0.001	< 0.001	0.210
Nasal	4.37 $\pm$ 4.67	3.75 $\pm$ 3.35	3.42 $\pm$ 3.09	< 0.001	< 0.001	< 0.001	< 0.001
Inferotemporal	4.48 $\pm$ 4.19	3.78 $\pm$ 3.04	3.43 $\pm$ 2.85	< 0.001	< 0.001	< 0.001	< 0.001
Inferonasal	5.23 $\pm$ 4.75	4.20 $\pm$ 3.23	3.97 $\pm$ 3.26	< 0.001	< 0.001	< 0.001	< 0.001
Peripheral	4.90 $\pm$ 3.94	4.04 $\pm$ 2.60	3.73 $\pm$ 2.48	< 0.001	< 0.001	< 0.001	< 0.001
Central	4.08 $\pm$ 4.18	3.76 $\pm$ 3.15	3.33 $\pm$ 2.68	< 0.001	< 0.001	0.001	< 0.001

*RMSE* root mean square error, *SD* standard deviation, *LR* linear regression, *LSTM* long short-term memory, *Bi-GRU* bidirectional gated recurrent unit.

*Significance level ɑ = 0.05.

^†^Significance level ɑ = 0.017.

**Figure 7:**
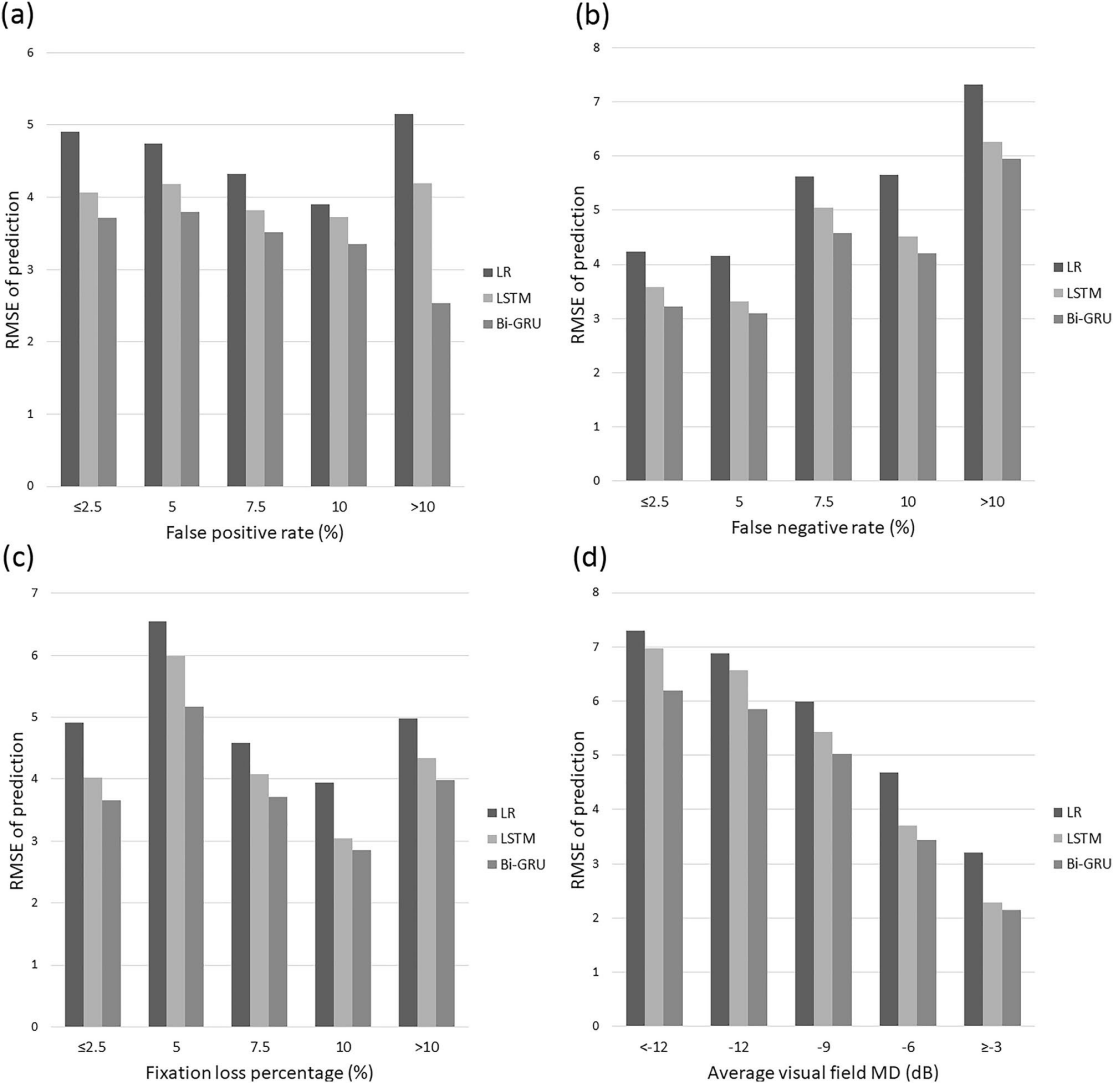
Average prediction error (RMSE) binned according to various factors. RMSE vs. **(a)** false positive rate, (**b**) false negative rate, (**c**) fixation loss percentage, and (**d**) visual field mean deviation (MD). Bi-GRU showed the lowest prediction error. LR = linear regression; LSTM = long short-term memory; Bi-GRU = bidirectional gated recurrent unit; RMSE = root mean squared error.

The mean RMSE values binned according to various factors are listed in Table 5 and Fig. 7. The prediction error was significantly lower for Bi-GRU than for the other two models in terms of the false-positive rate, false-negative rate, and fixation loss percentage (*p* ≤ 0.025). As the visual field MD increased, the RMSE prediction errors of all three models decreased.

**Table 5:** Mean prediction error (RMSE) binned according to reliability indices and visual field mean deviation.

	Prediction error (RMSE, dB), mean ± SD			Number of eyes	*p* value*	*p* value^†^		
	LR	LSTM	Bi-GRU			Bi-GRU vs. LSTM	Bi-GRU vs. LR	LR vs. LSTM
Prediction error vs. false positive rate (FPR, %)								
FPR ≤ 2.5	4.90 ± 4.32	4.06 ± 2.65	3.71 ± 2.44	797	< 0.001	< 0.001	< 0.001	< 0.001
2.5 < FPR $\le$ 5.0	4.74 ± 3.25	4.18 ± 2.69	3.80 ± 2.53	258	< 0.001	< 0.001	< 0.001	< 0.001
5.0 < FPR $\le$ 7.5	4.32 ± 2.52	3.82 ± 2.38	3.52 ± 2.18	72	< 0.001	< 0.001	< 0.001	0.007
7.5 < FPR $\le$ 10.0	3.90 ± 2.28	3.73 ± 2.13	3.35 ± 1.94	57	< 0.001	< 0.001	0.001	0.321
FPR > 10	5.15 ± 3.19	4.19 ± 2.53	3.84 ± 2.33	88	< 0.001	< 0.001	< 0.001	< 0.001
Prediction error vs. false negative rate (FNR, %)								
FNR ≤ 2.5	4.23 ± 3.88	3.58 ± 2.49	3.22 ± 2.21	766	< 0.001	< 0.001	< 0.001	< 0.001
2.5 < FNR $\le$ 5.0	4.16 ± 2.92	3.32 ± 1.79	3.10 ± 1.59	155	< 0.001	< 0.001	< 0.001	< 0.001
5.0 < FNR $\le$ 7.5	5.62 ± 3.02	5.05 ± 2.31	4.57 ± 2.06	109	< 0.001	< 0.001	< 0.001	0.007
7.5 < FNR ≤ 10.0	5.65 ± 2.91	4.52 ± 2.05	4.20 ± 1.89	91	< 0.001	< 0.001	< 0.001	< 0.001
FNR > 10	7.32 ± 4.67	6.26 ± 3.03	5.94 ± 3.08	151	< 0.001	< 0.001	< 0.001	< 0.001
Prediction error vs. fixation loss percentage (FLP, %)								
FLP ≤ 2.5	4.91 ± 4.88	4.03 ± 2.74	3.66 ± 2.52	518	< 0.001	< 0.001	< 0.001	< 0.001
2.5 < FLP ≤ 5.0	6.54 ± 2.99	5.99 ± 2.20	5.17 ± 2.06	13	0.042	0.002	0.025	0.422
5.0 < FLP ≤ 7.5	4.59 ± 2.87	4.08 ± 2.61	3.71 ± 2.38	175	< 0.001	< 0.001	< 0.001	0.001
7.5 < FLP ≤ 10.0	3.95 ± 3.44	3.05 ± 2.19	2.86 ± 2.10	131	< 0.001	< 0.001	< 0.001	< 0.001
FLP > 10	4.98 ± 2.93	4.34 ± 2.50	3.98 ± 2.34	435	< 0.001	< 0.001	< 0.001	< 0.001
Prediction error vs. average visual field mean deviation (MD, dB)								
MD < − 12	7.30 ± 4.56	6.98 ± 2.49	6.20 ± 2.69	230	< 0.001	< 0.001	< 0.001	0.173
− 12 ≤ MD < − 9	6.88 ± 2.86	6.57 ± 2.04	5.85 ± 2.10	80	< 0.001	< 0.001	< 0.001	0.229
− 9 ≤ MD < − 6	5.99 ± 2.44	5.43 ± 1.90	5.02 ± 1.80	142	< 0.001	< 0.001	< 0.001	0.002
− 6 ≤ MD < − 3	4.68 ± 3.97	3.70 ± 1.94	3.44 ± 1.72	278	< 0.001	< 0.001	< 0.001	< 0.001
− 3 ≤ MD	3.20 ± 3.12	2.28 ± 1.28	2.14 ± 1.17	542	< 0.001	< 0.001	< 0.001	< 0.001

*RMSE* root mean square error, *SD* standard deviation, *LR* linear regression, *LSTM* long short-term memory, *Bi-GRU* bidirectional gated recurrent unit, *FPR* false positive rate, *FNR* false negative ratio, *FLP* fixation loss percentage, *MD* mean deviation.

*Significance level ɑ = 0.05.

^†^Significance level ɑ = 0.017.

**Figure 8:**
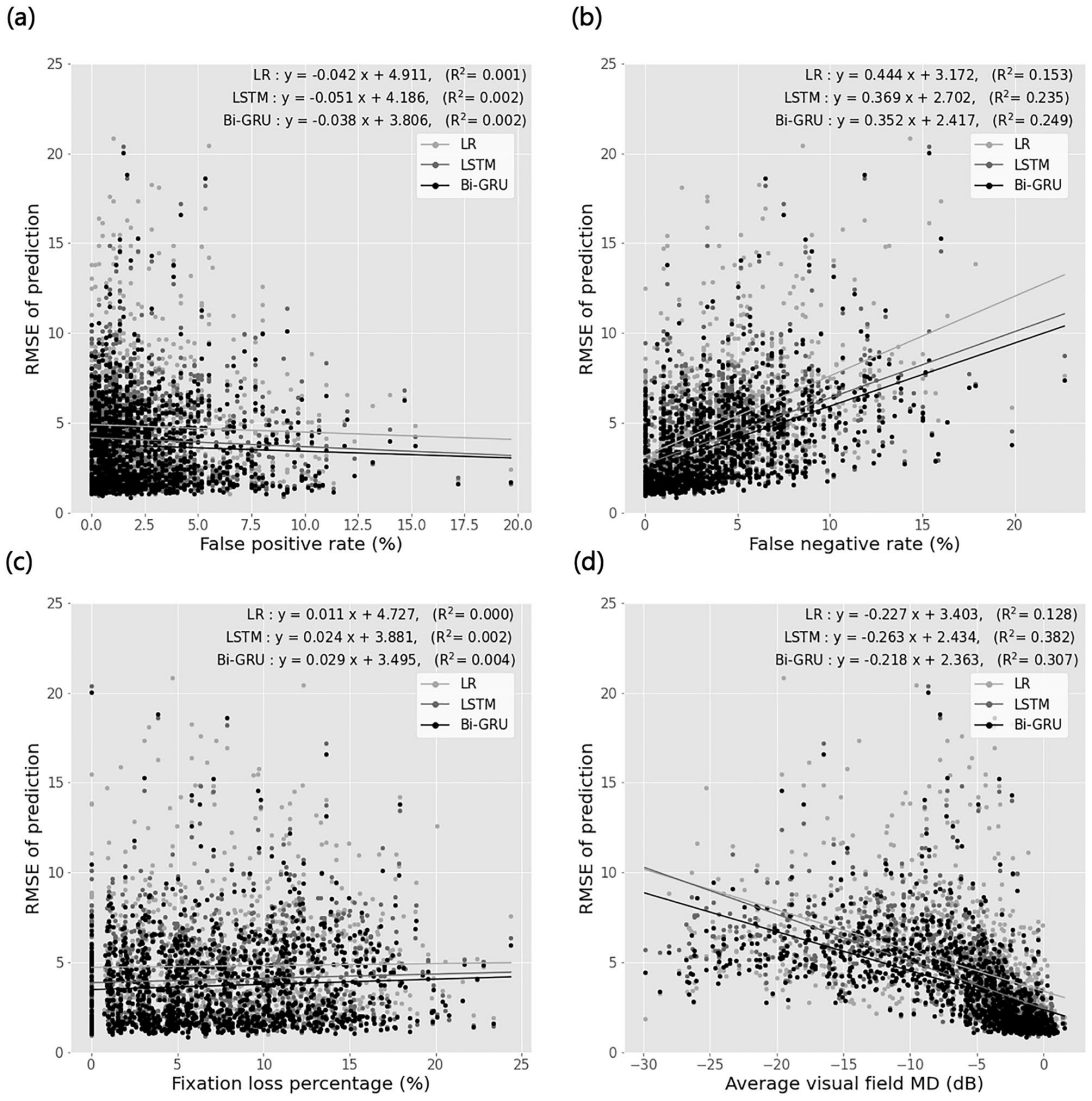
Linear regression analysis between prediction error (RMSE) and various factors. RMSE vs. (**a**) false positive rate, (**b**) false negative rate, (**c**) fixation loss percentage, and (**d**) visual field mean deviation (MD). LR = linear regression; LSTM = long short-term memory; Bi-GRU = bidirectional gated recurrent unit; RMSE = root mean squared error.

The correlation coefficients and LR analyses between the prediction error and various factors are presented in Table 6 and Fig. 8. For all models, RMSE was positively correlated with the false-negative rate and fixation loss percentage, whereas it was negatively correlated with visual field MD (all *p* ≤ 0.029) (Fig. 8).

**Table 6:** Correlation coefficients and linear regression analyses between prediction error and reliability, and between prediction error and visual field mean deviation.

	Correlation coefficients		Linear regression analysis			
	Spearman’s rho	*p* value	Slope	Intercept	${R}^{2}$	*p* value
Prediction error vs false positive rate						
LR	− 0.025	0.367	− 0.042	4.911	0.001	0.329
LSTM	− 0.053	0.060	− 0.051	4.186	0.002	0.078
Bi-GRU	− 0.042	0.135	− 0.038	3.806	0.002	0.151
Prediction error vs false negative rate						
LR	0.574	< 0.001	0.444	3.172	0.153	< 0.001
LSTM	0.543	< 0.001	0.369	2.702	0.235	< 0.001
Bi-GRU	0.558	< 0.001	0.352	2.417	0.249	< 0.001
Prediction error vs fixation loss percentage						
LR	0.083	0.003	0.011	4.727	< 0.001	0.626
LSTM	0.061	0.029	0.024	3.881	0.002	0.101
Bi-GRU	0.076	0.006	0.029	3.495	0.004	0.032
Prediction error vs average visual field mean deviation						
LR	− 0.671	< 0.001	− 0.227	3.403	0.128	< 0.001
LSTM	− 0.773	< 0.001	− 0.263	2.434	0.382	< 0.001
Bi-GRU	− 0.755	< 0.001	− 0.218	2.363	0.307	< 0.001

*LR* linear regression, *LSTM* long short-term memory, *Bi-GRU* bidirectional gated recurrent unit.

## Discussion

To the best of our knowledge, this study is the first to utilize the Bi-GRU architecture for predicting visual field loss. We compared the prediction of visual field loss using the Bi-GRU, LR, and LSTM models. The Bi-GRU model demonstrated the highest predictive accuracy among the three models. The overall prediction errors (RMSEs) of the LR, LSTM, and Bi-GRU models were 4.81 ± 3.89, 4.06 ± 2.61, and 3.71 ± 2.42 dB, respectively. The RMSE significantly differed between Bi-GRU and the other models (*p* < 0.001).

In the six sectors of the visual fields according to optic nerve head anatomy, as well as the central and peripheral visual field areas, Bi-GRU exhibited superior performance compared with the other two models (all *p* < 0.001).

The predictive performance was negatively correlated with the false-negative rate and fixation loss percentage in all three models; however, Bi-GRU was least affected by reliability indices. A decrease in MD was associated with lower prediction performance in all three models. The RMSE was lowest for Bi-GRU among the three models; Bi-GRU performed better even in patients with advanced glaucoma.

Several studies have used artificial intelligence to detect glaucoma and its progression. Asaoka et al.^23^ built a deep feed-forward neural network to detect preperimetric glaucoma. The area under the receiver operating characteristic curve (AUROC) of the model was 92.6%, indicating better performance than other machine learning methods (e.g., random forest, gradient boosting, support vector machine, and neural network). Although that study was the first to use deep learning for the evaluation of preperimetric glaucoma, only a small quantity of data from preperimetric visual fields of patients with glaucoma (53 eyes) were analyzed. Elze et al.^24^ classified visual fields into 16 archetypes and found that the archetypes were closely correlated with the clinical features of glaucoma^25^. However, these studies classified visual fields, rather than predicting visual field changes. Yousefi et al.^26^ compared various machine learning algorithms in terms of detecting glaucoma progression, using the retinal nerve fiber layer on optical coherence tomography and the MD and pattern standard deviation on visual field examination as input. The random forest classifier showed the best performance, with an AUROC of 0.88. Wang et al.^4^ assessed the predictive ability for visual field changes using archetypes; they found that the mean hit and correct rejection rates were 0.77 and 0.77, suggesting that the predictive ability of the archetype approach was higher than the abilities of other methods, such as MD slope, advanced glaucoma intervention study scoring, collaborative initial glaucoma treatment study scoring, and the permutation of pointwise linear regression. However, unlike our study, previous studies did not predict visual field changes.

Dixit et al.^14^ found that the progression of visual field changes using a deep learning algorithm based on LSTM architecture could be predicted with an accuracy of 91–93%. The AUROC was 0.89–0.93 when using multiple visual field examinations and baseline clinical data as input. Additionally, the use of clinical data to supplement the visual field data led to improved model performance. Murata et al.^5^ found that variational Bayes linear regression more accurately predicted the progression of visual field changes in patients with glaucoma, compared with conventional least-squares LR. Wen et al.^6^ used Cascade-Net, a type of convolutional neural network architecture, to predict future Humphrey visual field findings using only a single visual field input. The models showed excellent predictive abilities; the overall PMAE and RMSE were 2.47 and 3.47 dB, respectively. The PMAE and RMSE of the Bi-GRU model were slightly higher than the PMAE and RMSE of the Cascade-Net model. However, this model may not reflect true progression because the authors used single visual field examination as input. Berchuck et al.^7^ used a generalized variational autoencoder algorithm to estimate progression rates and predict future visual fields. The overall MAE was 1.89–2.33 dB, comparable with the MAE of our model. Park et al.^13^ used an RNN to predict the sixth visual field examination; they found that the RMSE was 4.31 ± 2.4 dB, indicating that RNN predicted future visual field better than LR.

In a previous study, we used the LSTM model to analyze time-sequential input consisting of visual field examinations^13^. In the present study, we built a deep learning architecture based on a Bi-GRU network. Both GRU and LSTM are variants of RNN, a state-of-the-art deep learning architecture that processes sequential data for sequence recognition and prediction^27^. Cho et al.^16^ presented a GRU architecture that allowed each recurrent unit to adaptively capture dependencies of different time scales. Both GRU and LSTM have recurrent units in sequence modeling. However, GRU has gating units that modulate the flow of information inside the unit without separate memory cells^8,12,16^. Chung et al.^12^ reported that GRU was comparable with LSTM for polyphonic music modeling and speech signal modeling. Khandelwal et al.^17^ found that GRU outperformed LSTM in terms of shorter computation time and lower word error rate for automatic speech recognition.

Conventional RNN only considers the previous context of training data. To overcome the limitations of a conventional RNN, Shuster et al.^20^ proposed a bidirectional RNN that considers both past and future input sequences to estimate the output vector. Several studies have shown that Bi-GRU outperforms LSTM^15,17,18^. Bi-GRU achieved the highest classification accuracy among deep neural network-based models for human identification based on electrocardiogram biometrics^15^.

In the present study, Bi-GRU exhibited better predictive performance than LR and LSTM for the entire visual field, as well as the central area; this area is important because the preservation of central visual function has a strong effect on quality of life in patients with glaucoma^28,29^. Bi-GRU was least affected by reliability indices. The false-negative rate and fixation loss affected visual field prediction in all models. However, there was poor correlation between fixation loss and visual field prediction, indicating a small effect of fixation loss. Previous studies showed that false-negative rates, but not fixation loss, were associated with visual field assessment^13,30,31^. Moreover, previous studies revealed that false-negative rates were the most common cause of unreliable visual field classification^32,33^.

Our study had several limitations. First, the study results cannot be fully generalized to patients with different degrees of glaucoma severity. The study included a greater number of patients with early glaucoma (MD >  − 6 dB) in the training and test datasets, compared with patients who had advanced glaucoma. Although this difference may have affected the performance of Bi-GRU model learning, it reflects the distribution of glaucoma severity observed in clinical practice.

Second, we did not include clinical data for training, in contrast to the work by Dixit et al.^14^ Future studies should improve deep learning architecture by adding clinical characteristics to the input data.

Third, we trained and tested the model using five consecutive visual field data elements as input. Glaucoma specialists recommend that at least five serial visual field examinations are used to detect glaucoma progression. The Glaucoma Progression Analysis included in the Humphrey Visual Field Analyzer requires at least five reliable visual field examinations and a follow-up period of 2 years^34^. Previous studies also used five visual field data elements as input to predict visual field progression in glaucoma^35,36^. Additionally, sequential pointwise LR was performed with at least four visual field examinations because regression analysis is unlikely to detect a trend when fewer data are available^37^. We predicted the sixth visual field examination using the previous five examinations to compare the predictive performances of Bi-GRU and LR models. Glaucoma requires lifelong periodic visual field examinations^38,39^. Thus, five consecutive visual field examinations over 3 years are not an excessively frequent number, and the prediction of subsequent examinations based on the initial five examinations may enhance patient convenience.

On further analysis, we predicted future visual field based on four consecutive visual field data elements using the Bi-GRU model. The mean prediction errors were 3.84 ± 2.48 and 2.91 ± 1.96 dB for RMSE and PMAE, respectively. Although there were statistically significant differences in prediction errors (both *p* < 0.001) between the models using five and four visual field data elements, the difference was not clinically significant.

Fourth, the model could only predict the sixth visual field examinations. Future studies should collect additional patient data with a greater number of visual field examinations and evaluate the performance of our model in terms of predicting the seventh through tenth visual field examinations, using the first five visual field examinations as input. However, our model can forcast visual fields at future time points. For example, the model can predict the visual fields at 4, 8, and 12 months after the fifth visual field examination.

In summary, a deep learning architecture using the Bi-GRU model, a variant of RNN, predicts future visual field examinations significantly better than the pointwise LR and LSTM models. The Bi-GRU model is less affected by the reliability indices of visual field input data. This model may facilitate decision-making by accurately predicting future visual field examinations in clinical practice, particularly for patients who experience difficulty with repeated examinations.

## Supplementary Information

Supplementary Information.

## Data Availability

The data generated or analyzed during this study are available from the corresponding author (J.R.P.) upon reasonable request.

## References

[1] Resnikoff, S. *et al.* Global data on visual impairment in the year 2002. *Bull. World Health Organization* 9 (2004). PMC2623053 15640920

[2] Weinreb RN, Aung T, Medeiros FA (2014). The pathophysiology and treatment of glaucoma: A review. JAMA.

[3] Henson, D. B., Chaudry, S., Artes, P. H., Faragher, E. B., & Ansons, A. Response variability in the visual field: Comparison of optic neuritis, glaucoma, ocular hypertension, and normal eyes. **41**, 5 (2000). 10670471

[4] Wang M (2019). An artificial intelligence approach to detect visual field progression in glaucoma based on spatial pattern analysis. Invest. Ophthalmol. Vis. Sci..

[5] Murata H, Araie M, Asaoka R (2014). A new approach to measure visual field progression in glaucoma patients using variational bayes linear regression. Invest. Ophthalmol. Vis. Sci..

[6] Wen JC (2019). Forecasting future Humphrey Visual Fields using deep learning. PLoS ONE.

[7] Berchuck SI, Mukherjee S, Medeiros FA (2019). Estimating rates of progression and predicting future visual fields in glaucoma using a deep variational autoencoder. Sci Rep.

[8] Salehinejad, H., Sankar, S., Barfett, J., Colak, E., Valaee, S. Recent advances in recurrent neural networks. 21.

[9] Liu, S., Yang, N., Li, M. & Zhou, M. A recursive recurrent neural network for statistical machine translation. In *Proceedings of the 52nd Annual Meeting of the Association for Computational Linguistics (Volume 1: Long Papers)* 1491–1500 (Association for Computational Linguistics, 2014). 10.3115/v1/P14-1140.

[10] Young T, Hazarika D, Poria S, Cambria E (2018). Recent trends in deep learning based natural language processing. IEEE Comput. Intell. Mag..

[11] Hochreiter S, Schmidhuber J (1997). Long short-term memory. Neural Comput..

[12] Chung, J., Gulcehre, C., Cho, K., & Bengio, Y. Empirical evaluation of gated recurrent neural networks on sequence modeling. (2014) 10.48550/ARXIV.1412.3555.

[13] Park K, Kim J, Lee J (2019). Visual field prediction using recurrent neural network. Sci Rep.

[14] Dixit A, Yohannan J, Boland MV (2021). Assessing glaucoma progression using machine learning trained on longitudinal visual field and clinical data. Ophthalmology.

[15] Lynn HM, Pan SB, Kim P (2019). A deep bidirectional gru network model for biometric electrocardiogram classification based on recurrent neural networks. IEEE Access.

[16] Cho, K. *et al.* Learning phrase representations using RNN encoder–decoder for statistical machine translation. In *Proceedings of the 2014 Conference on Empirical Methods in Natural Language Processing (EMNLP)* 1724–1734 (Association for Computational Linguistics, 2014). 10.3115/v1/D14-1179.

[17] Khandelwal, S., Lecouteux, B. & Besacier, L. Comparing GRU and LSTM for Automatic Speech Recognition. 7.

[18] Li X (2022). Time-series production forecasting method based on the integration of Bidirectional Gated Recurrent Unit (Bi-GRU) network and Sparrow Search Algorithm (SSA). J. Petrol. Sci. Eng..

[19] Darmawahyuni A, Nurmaini S, Rachmatullah MN, Firdaus F, Tutuko B (2021). Unidirectional-bidirectional recurrent networks for cardiac disorders classification. TELKOMNIKA.

[20] Schuster M, Paliwal KK (1997). Bidirectional recurrent neural networks. IEEE Trans. Signal Process..

[21] Pascanu, R., Gulcehre, C., Cho, K. & Bengio, Y. How to construct deep recurrent neural networks. (2013). 10.48550/ARXIV.1312.6026.

[22] Garway-Heath, D. F., Poinoosawmy, D., Fitzke, F. W. & Hitchings, R. A. Mapping the visual field to the optic disc in normal tension glaucoma eyes. **107**, 7 (2000). 10.1016/s0161-6420(00)00284-0 11013178

[23] Asaoka R, Murata H, Iwase A, Araie M (2016). Detecting preperimetric glaucoma with standard automated perimetry using a deep learning classifier. Ophthalmology.

[24] Elze T (2015). Patterns of functional vision loss in glaucoma determined with archetypal analysis. J. R. Soc. Interface..

[25] Cai S (2017). Clinical correlates of computationally derived visual field defect archetypes in patients from a glaucoma clinic. Curr. Eye Res..

[26] Yousefi S (2018). Detection of longitudinal visual field progression in glaucoma using machine learning. Am. J. Ophthalmol..

[27] Bengio Y, Simard P, Frasconi P (1994). Learning long-term dependencies with gradient descent is difficult. IEEE Trans. Neural Netw..

[28] Johnson CA, Nelson-Quigg JM (1993). A prospective three-year study of response properties of normal subjects and patients during automated perimetry. Ophthalmology.

[29] Katz J, Sommer A, Witt K (1991). Reliability of visual field results over repeated testing. Ophthalmology.

[30] Murata H (2013). Identifying areas of the visual field important for quality of life in patients with glaucoma. PLoS ONE.

[31] Abe RY (2016). The impact of location of progressive visual field loss on longitudinal changes in quality of life of patients with glaucoma. Ophthalmology.

[32] Rao HL (2015). Role of visual field reliability indices in ruling out glaucoma. JAMA Ophthalmol.

[33] Raman, P., Khy Ching, Y., Sivagurunathan, P. D., Ramli, N. & Mohd. Khalid, K. H. The Association between visual field reliability indices and cognitive impairment in glaucoma patients. *J. Glaucoma* **28**, 685–690 (2019). 10.1097/IJG.0000000000001269 31033782

[34] Casas-Llera P (2009). Visual field index rate and event-based glaucoma progression analysis: Comparison in a glaucoma population. Br. J. Ophthalmol..

[35] Crabb DP, Fitzke FW, McNaught AI, Edgar DF, Hitchings RA (1997). Improving the prediction of visual field progression in glaucoma using spatial processing. Ophthalmology.

[36] Bengtsson B (2009). Prediction of glaucomatous visual field loss by extrapolation of linear trends. Arch Ophthalmol.

[37] Nouri-Mahdavi K (2007). Comparison of methods to predict visual field progression in glaucoma. Arch Ophthalmol.

[38] European Glaucoma Society Terminology and Guidelines for Glaucoma, 5th Edition. *Br. J. Ophthalmol.* **105**, 1–169 (2021). 10.1136/bjophthalmol-2021-egsguidelines 34675001

[39] Prum BE (2016). Primary open-angle glaucoma preferred practice pattern® guidelines. Ophthalmology.

