# Supplementary material for: Visual field prediction using a deep bidirectional gated recurrent unit network model
Source: Sci Rep. 2023 Jul 10;13:11154. doi: 10.1038/s41598-023-37360-1 (PMC10333213; doi:10.1038/s41598-023-37360-1)
Supplement: Supplementary file 1 — Supplementary Information. [file 41598_2023_37360_MOESM1_ESM.pdf]

Visual Field Prediction using a Deep Bidirectional Gated Recurrent Unit Network Model

Hwayeong Kim[1†], Jiwoong Lee[1,2†], Sangwoo Moon[1], Sangil Kim[3], Taehyeong Kim[3], Sang Wook Jin[4], Jung Lim Kim[5], Jonghoon Shin[6], Seung Uk Lee[7], Geunsoo Jang[3], Yuanmeng Hu[3], Jeong Rye Park[8,*]

[1]*Department of Ophthalmology, Pusan National University College of Medicine, Busan, Korea*

[2]*Biomedical Research Institute, Pusan National University Hospital, Busan, Korea*

[3]*Department of Mathematics, Pusan National University, Busan, Republic of Korea*

[4]*Department of Ophthalmology, Dong-A University College of Medicine, Busan, Korea*

[5]*Department of Ophthalmology, Busan Paik Hospital, Inje University College of Medicine, Busan, Korea*

[6]*Department of Ophthalmology, Pusan National University Yangsan Hospital, Pusan National University School of Medicine, Yangsan, Korea*

[7]*Department of Ophthalmology, Kosin University College of Medicine, Busan, Korea*

[8]Department of Mathematics, Kyungpook National University, Daegu, Republic of Korea

†These authors contributed equally to this work as the first authors.

Corresponding Author: Jeong Rye Park, Ph.D.

Corresponding Author: Jeong Rye Park, Ph.D. Department of Mathematics, Kyungpook National University, 80, Daehak-ro, Buk-gu, Daegu, Republic of Korea, 41566 E-mail: parkjr@knu.ac.kr

Supplementary Figure S1. (**a**) The structures of one LSTM cell and (**b**) one GRU cell.

In both the LSTM and GRU cells, the orange block represents the sigmoid function, x represents the cross product, and (+) represents concatenation.

(a)

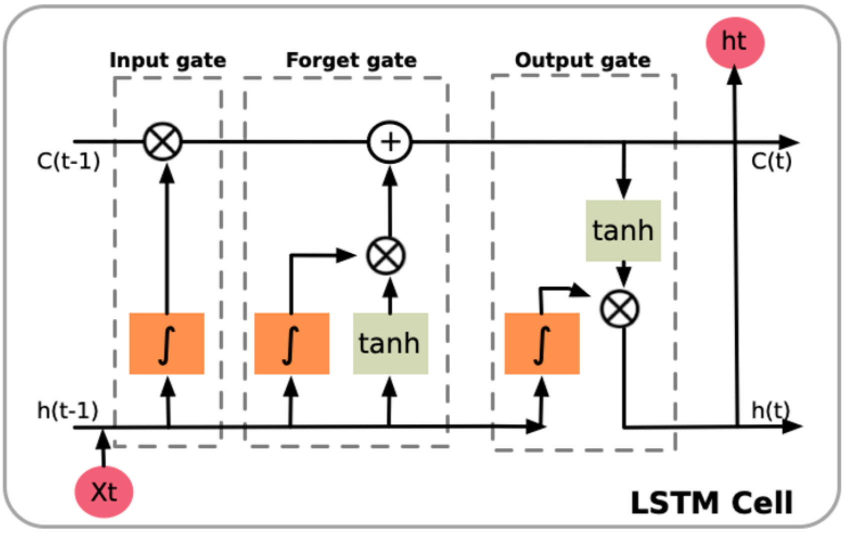

(b)

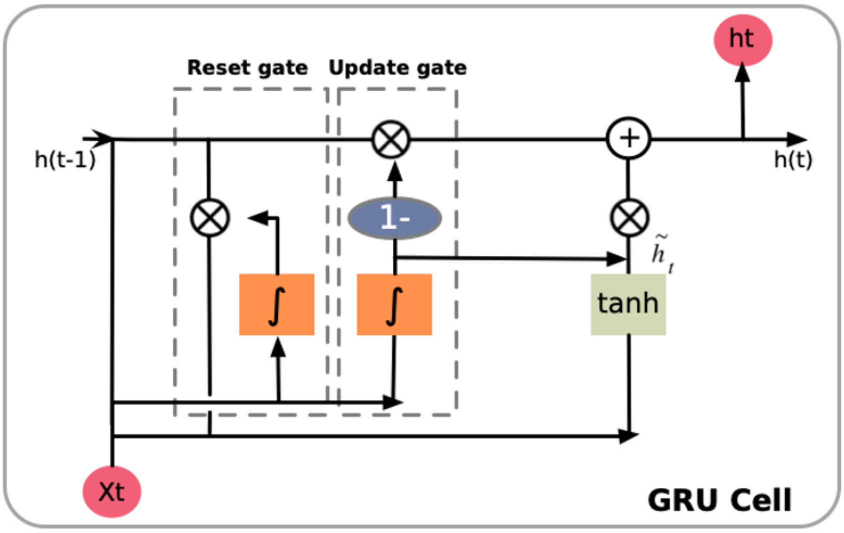

LSTM = long short-term memory; GRU = gated recurrent unit
